# Oro-Pharyngeal Candidiasis in Two Dengue Patients

**DOI:** 10.7759/cureus.52627

**Published:** 2024-01-20

**Authors:** Drupad Das, Shiv N Sahu, Prasan K Panda, Madhusmita Panda

**Affiliations:** 1 Internal Medicine, All India Institute of Medical Sciences, Rishikesh, Rishikesh, IND; 2 Family Medicine, Midland Health, Midland, USA

**Keywords:** mutant era, oral thrush, oral candidiasis, hyperglycemia, dengue hemorrhagic fever (dhf)

## Abstract

Dengue, a prevalent arboviral disease, has witnessed a resurgence in India, with outbreaks frequently reported. However, dengue-associated oral (oro-pharyngeal) candidiasis (DAOC) was never reported. We present two severe dengue cases with oral/oro-pharyngeal pseudomembranous candidiasis. Case 1 of a young man without any comorbidities or abuse or immunosuppression presented with fever, headache, altered sensorium, throat pain on recovery, and laboratory reports confirmed dengue with leukopenia, thrombocytopenia, and severe hepatic involvement with oro-pharyngeal candidiasis. Similarly, case 2 of a middle-aged man with a history of smoking and diabetes presented with fever, gum bleeding, and throat pain, later confirmed to be dengue NS1 positive with thrombocytopenia, and mild-moderate hepatic involvement along with oral-oro-pharyngeal candidiasis. Both cases showed improvement with conservative management and oral nystatin suspension. These cases prompt consideration of superadded candida infections in dengue patients, emphasizing the need for further study and clinical vigilance.

## Introduction

Dengue is one of the most common arboviral diseases of humans. The resurgence of dengue or dengue outbreaks has been frequently reported from different parts of India. WHO classification guidelines for dengue divide it into dengue fever, dengue hemorrhagic fever without shock or with shock (DSS) and expanded dengue syndrome. Expanded dengue syndrome is a new entity added to the classification to incorporate a wide spectrum of unusual manifestations and involvement of various organ systems, including the gastrointestinal tract, hepatic, neurological, pulmonary, and renal systems. The endothelium is the primary target of immunopathogenesis in dengue fever. The hallmark of this is increased vascular permeability and coagulation disorders. While severe viral infections may lead to superadded bacterial or fungal infections, cases of dengue-associated oral (oro-pharyngeal) candidiasis (DAOC) are not extensively reported [1].

Generally, Candida is part of normal commensal microflora and 30%-60% of the adult population carry it in the oral cavity [2]. Oral thrush or acute pseudomembranous candidiasis is the most common form of oral candidiasis. Other forms of oral candidiasis may present as chronic hyperplastic candidiasis, acute or chronic erythematous candidiasis, angular cheilitis, median rhomboid glossitis, and linear gingival erythema [3]. It is generally seen in immunosuppressed states, which can be either local or systemic like extremes of age (elderly or newborns), HIV/AIDS, and chronic systemic or local steroid and antibiotic use. Few case reports are published that show candida infection in the form of dissemination and sinusitis after severe dengue due to endothelial dysfunction caused by the dengue virus itself or by immune reaction [3-5].

Here, we are presenting two cases of severe dengue fever - case 1 having no prior risk factor and case 2 having risk factor (diabetes) who presented to the hospital with oral pseudomembranous candidiasis.

## Case presentation

Case 1

A 30-year-old man, resident of Uttarakhand, security officer by occupation presented to the emergency department with a history of acute onset high-grade fever for the last seven days. He was associated with mild to moderate intensity holocranial headache and hiccups for the last five days; and acute onset, continuous, dull aching, right hypochondrial pain on a log scale of 4 out of 10 for the last four days. He also complained of dizziness with one episode of fall without loss of consciousness or seizure episode three days back. He was admitted to a hospital and had a total platelet count of 54,000/dL with Dengue NS1 positivity. In the hospital, he developed agitation, aggressive behavior, and confusion not recognizing family members, followed by drowsiness and aphasia. For these complaints, he was referred to our tertiary care hospital. There was no history of vomiting, rash, diplopia, blurring of vision, seizure episode, or any bleeding manifestation. There was no history of recurrent fever, cough, or any recurrent infections in the past to suggest any immunocompromised state neither he had any history suggestive of vector-borne diseases. Social history was negative for smoking, alcohol, illicit drugs, or high-risk behavior. Documentation of the last hospital admission did not show the administration of any antibiotics or steroids.

**Figure 1:**
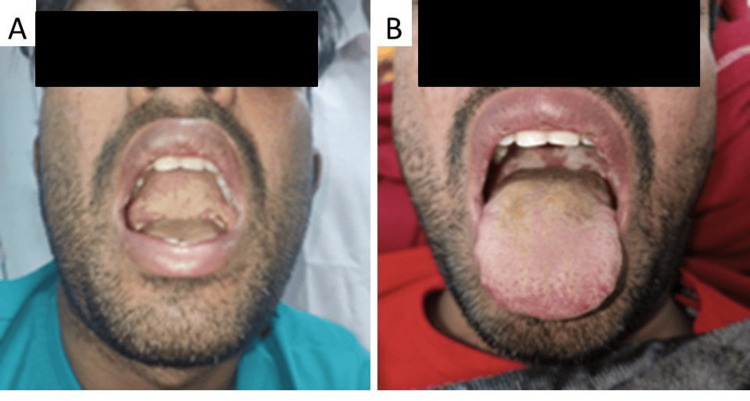
Oro-pharyngeal candidiasis Case 1 has multiple creamy white lesions over the posterior pharynx (A, B).

On physical examination, the patient had sinus bradycardia of 48 heart rate per minute with normal blood pressure, a random blood glucose of 243 mg/dL, had icterus and was conscious but disoriented to time, place, and person, and had hoarseness of voice. No signs of meningeal irritation were noted, and other neurological examinations were normal. On respiratory system examination, he had inspiratory crackles over bilateral infra-axillary and infra-scapular areas. Multiple creamy white lesions were noted on the posterior oro-pharyngeal wall and palate (Figures 1A, 1B). The patient had mild tenderness on the right hypochondriac region without organomegaly.

His blood investigation showed worsening thrombocytopenia (38,000 cells/mm^3^), leukopenia (2,600 cells/mm^3^), and hematocrit of 37.2%. Hydration with intravenous fluid was continued and gradually his sensorium improved on the next day. Peripheral smear showed no atypical cell or blast. Prothrombin time was within normal (14.6 seconds). Serum glutamic oxaloacetic transaminase (SGOT) was 2,912 units/liter serum glutamic pyruvic transaminase (SGPT) was 1,712 units/liter, total bilirubin of 7.3 mg/dL and direct bilirubin of 4.5 mg/dL, alkaline phosphatase was 150 Units/liter. The renal function test was within normal limits. Microscopy of oro-pharyngeal swabs showed budding yeast cells, and culture of the same grew candida. Cardiac biomarkers (CPK-MB - 11 IU/L, troponin-T - 0.01 ng/mL) were within normal limits. Tropical fever workup showed dengue NS1 positive (ELISA method). Anti-HIV, anti-HCV, and HBsAg were negative. HBA1C came out to be 7.1% and fasting C-peptide was within normal (3.44 ng/mL). USG whole abdomen showed mild hepatomegaly (16.8cm). Fundoscopy showed no evidence of papilledema, and indirect laryngoscopy showed normally functioning vocal cords, with mild congestion.

Severe dengue fever was kept as a primary diagnosis. There was no other endocrinopathy or hearing loss in the patient. Although diabetes mellitus is itself an immunodeficiency condition, it was not severe in this case. The possibility of primary immunodeficiency cannot be ruled out, but no history of recurrent infections was there.

The sensorium of the patient improved gradually over the next one to two days. Liver function tests also improved, and platelet count started improving with conservative management without any antibiotics. For oral candidiasis, nystatin ointment was used, and it improved significantly. Hoarseness also started improving with voice rest and gargling with chlorohexidine mouthwash.

Case 2

**Figure 2:**
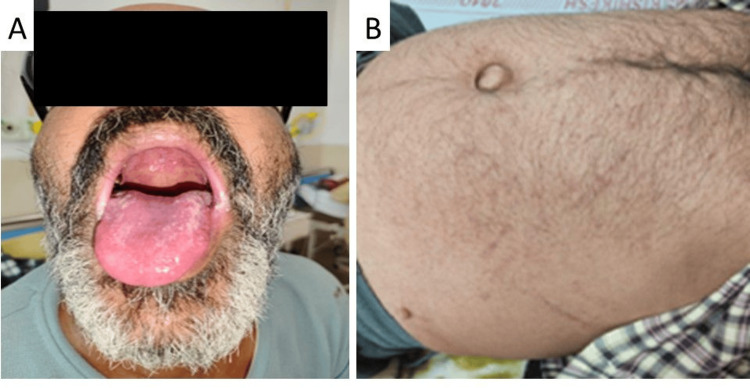
Oro-pharyngeal candidiasis and purpuric spots over abdomen Case 2 has similar lesions over the dorsum of the tongue and post pharynx (A) and erythematous skin rashes all over the body (B, abdomen shown).

A 43-year-old man, a resident of Uttar Pradesh, driver by occupation, a smoker for 25 years, and occasional alcohol consumer, having a previous history of dengue fever six years back, known patient of diabetes mellitus, presented with a history of acute onset intermittent, high-grade fever associated with chills during the last seven days followed by mild to moderate epigastric pain and two episodes of gum bleeding. He also noticed yellowish discoloration of eyes for three to four days, and throat pain for two days. On examination, the patient was hemodynamically stable with a random blood sugar of 180 mg/dL and had multiple ecchymoses over the arm and abdomen, icterus, and multiple creamy white patches over the tongue, posterior oro-pharyngeal wall and palate (Figures 2A, 2B). Tenderness was noted on the right hypochondriac region; on respiratory system examination, the patient had inspiratory crackles over bilateral infra-axillary and infra-scapular areas. The rest of the systemic examination was unremarkable.

Baseline blood investigation showed hematocrit of 39.7%, thrombocytopenia of 19,000/mm^3^, and total leukocyte count of 6,700/mm^3^; liver function test showed total bilirubin of 3.56 mg/dL, direct bilirubin of 2.00 mg/dL, SGPT of 450 Units/liter and SGOT of 633 Units/liter, with alkaline phosphatase of 559 Units/liter. The renal function test was within normal limits. A tropical fever workup came positive for dengue NS1 (ELISA method), and viral markers (anti-HCV, anti-HIV I/II, and HBsAg) were non-reactive. Serum HBA1C level was 10%. Fundoscopy showed no evidence of hypertensive or diabetic retinopathy, and urine routine microscopy showed no evidence of proteinuria. Abdominal ultrasonography showed grade II fatty liver with coarse liver echotexture without any sign of portal hypertension or dilatation of biliary radicle. Upper gastrointestinal endoscopy showed evidence of oro-pharyngeal candidiasis but not esophageal involvement or any varices there.

The patient improved with conservative management and transfusion of random donor platelets. Oral thrush improved with nystatin suspension, 4 mL four times a day. Over the next two weeks, the patient reported a complete resolution of throat pain. Blood glucose was optimized using subcutaneous regular insulin (10U three times a day) in the outpatient setting.

## Discussion

Both of the cases discussed here had clinical features of dengue fever with NS1 positivity and oro-pharyngeal candidiasis. Detection of NS1 antigen has a sensitivity of 60%-80% based on different diagnostic kits but has a good specificity of 97%-100% across different manufacturers [6]. There are many reported bloodstream infections in dengue such as *Streptococcus, Escherichia* ​​​​​*coli, Salmonella, Shigella, Klebsiella, Enterococcus, Moraxella, Staphylococcus, Haemophilus, Candida, Mycobacterium, Mycoplasma, or Herpesviruses* [7]. Candida is a commensal of the skin and intestinal tract. There is evidence of intestinal mucosal injury in dengue that leads to the transfer of organisms into the bloodstream resulting in candidemia [3,8]. Similarly, there are reports of dengue-associated invasive fungal sinusitis [5]. However, what causes dengue to present with this invasive candidiasis is not known.

Marrow suppression also occurs within three to four days after infection with the dengue virus, leading to a decrease in the number of neutrophils, which reaches its lowest point by the fifth or sixth day [9]. It is suggested that marrow shutdown can happen even if only a small proportion of cells, including ARCs, are infected with the dengue virus, as these infected cells send signals to halt the proliferation of all other cells. This early warning of infection results in hematopoietic stem cells, which are resistant to infection, ceasing their division into early blast cells, which are susceptible to infection. As a result, the bone marrow conserves the hematopoietic progenitor cell pool, ensuring a swift recovery once the infection is cleared [10]. Serious bacterial infections are not reported in these neutropenic conditions as they recover very fast and neither any study has been done for the requirement of prophylactic antibiotics.

Another interesting observation in the first case, he was diagnosed with first-time diabetes with HbA1c > 6.5% without any past or present history suggestive of diabetes as if this dengue precipitated the diagnosis of diabetes. Since we do not have any literature to establish dengue is the cause of diabetes, we have to correlate it with surrounding evidence such as SARS-CoV-2 infection was found to be associated with hyperglycemia during the COVID-19 era [11].

In the COVID-19 era, it was established interaction of dengue virus and SARS-CoV-2 in terms of their antibody-dependent enhancement and cross-reactivity resulted in capillary leakage, thrombocytopenia, coagulopathy, and cytokine storm in both diseases’ presentation [12,13]. Also, there is a proven fact of post-COVID-19 fungal diseases, but post-dengue fungal diseases are not much known. Probably change in the mutation of viruses resulting in different patterns of manifestation and host reactivity leads to superadded or activated viral infection as seen in these two cases. In the future, we can only explain the interaction between the dengue virus and candida. These case reports raise the question of the requirement for further study and demand clinical vigilance to look for superadded infection in patients with dengue fever when an outbreak occurs.

## Conclusions

Similar to COVID, dengue can cause or precipitate hyperglycemia, diabetes, or diabetes-associated candidiasis de novo. In this viral mutant era, each viral infection is to be monitored closely for expandable presentations. Oro-pharyngeal candidiasis, as associated with these two dengue cases, is interestingly observed and must be searched by clinicians during the dengue season ahead.
